# Enzymatic and Synthetic Routes of Castor Oil Epoxidation

**DOI:** 10.3390/polym15112477

**Published:** 2023-05-27

**Authors:** Juliana A. S. Montenegro, Andreas Ries, Ingridy D. S. Silva, Carlos B. B. Luna, Antônia L. Souza, Renate M. R. Wellen

**Affiliations:** 1 Materials Engineering Department, Federal University of Paraíba, João Pessoa 58051-900, Brazil; julianamontenegro14@gmail.com; 2 Multidisciplinary Center for Technological Investigations, National University of Asunción, San Lorenzo University Campus, San Lorenzo 111421, Paraguay; ries750@yahoo.com.br; 3 Academic Unit of Materials Engineering, Federal University of Campina Grande, Campina Grande 58249-140, Brazil; ingrydy.dayane@gmail.com (I.D.S.S.); brunobarretodemaufcg@hotmail.com (C.B.B.L.); 4 Chemistry Department, Federal University of Paraíba, João Pessoa 58051-900, Brazil; antonia_lucia@yahoo.com.br

**Keywords:** epoxidation, castor oil, lipase enzyme, FTIR, ^1^H-NMR

## Abstract

Epoxidation of castor oil in synthetic and enzymatic routes was carried out in order to promote a system with less environmental impact. The epoxidation reactions of castor oil compounds upon addition of lipase enzyme with and without acrylic immobilization and with reaction times of 24 and 6 h, as well as the synthetic compounds upon addition of Amberlite resin and formic acid, were investigated using Fourier transform infrared spectroscopy (FTIR) and nuclear magnetic resonance in hydrogen molecules (^1^H-NMR). The analysis indicated that the enzymatic reactions (6 h) and synthetic reactions provided a conversion from 50 to 96% and epoxidation from 25 to 48%, resulting from peak stretching and signal disintegration in the hydroxyl region due to the appearance of H_2_O in the interaction of peracid with catalyst. In systems without toluene, a dehydration event with a peak absorbance of 0.02 AU, indicating a possible vinyl group at 2355 cm^−1^ in enzymatic reactions without acrylic immobilization, was observed and resulted in a selectivity of 2%. In the absence of a solid catalyst, an unsaturation conversion of castor oil above 90% was achieved; however, this catalyst is necessary for the epoxidation to take place, whereas the lipase enzyme becomes able of epoxidizing and dehydrating the castor oil upon changing the time or reaction system. The conversation from 28 to 48% of solid catalysts (Amberlite and lipase enzyme) displays their importance to the instauration conversion of castor oil into oxirane rings.

## 1. Introduction

Epoxy resins, based on vegetable oils such as soy, linseed, and castor bean, have currently been receiving more attention from the scientific community. Researchers have synthesized new resins, replacing non-biodegradable, petroleum-based resins with products based on natural sources [1,2]. Among vegetable oils, castor oil has become a promising chemical product that can be epoxidized, as its main reactive component is 12-hydroxy-9-cis-octadecenoic acid, or ricinoleic acid [3,4].

The castor oil epoxidation mechanism is based on the principle of double bond replacement by the oxirane ring, a process which is established in ricinoleic acid as it represents 90% of castor oil composition [5]. Only a double bond is considered in the conversion [6] which, in addition to the presence of a hydroxyl group, promotes the oil’s natural functionality by providing cross-linking and dehydration to the material [4].

Vegetable oil epoxidations rely on several methodologies already applied in the academy and industry which use sundry reagents [7], such as epoxidations with organic and inorganic peroxides [8,9], acid catalysts [10], or enzymes [11]. Due to their hazardous effects, alternative catalysts with clean epoxidation routes have been researched among natural components to replace catalysts such as ion exchange resins [12,13] and performic acid [7,8,9,10,11,12,13,14]. Since neutralization and removal of the final product is required, strong mineral acids, which are used as catalysts, are being replaced by enzymes, especially lipase, a class of enzymes used in biocatalysis [15]. This promotes the chemoenzymatic epoxidation of vegetable oils [16,17], which provides reactions with enantiomeric selectivity, resulting in clean compounds at an industrial scale [18].

Most enzymes become less active and stable in organic solvents [19]. Therefore, the enzymes used in the literature for the chemoenzymatic epoxidation of vegetable oils are immobilized using low-cost supports, such as polyacrylamide as used by Knight et al. [20]. The lipase immobilization from Fusarium solani FS1, with retention of 53% of enzymatic activity, increased specificity, selectivity, and improved stability of its structure, allows for its recovery and reuse [21,22,23].

The enzymatic catalysts most used in scientific research are Candida antarctica lipase B (CALB), which can be immobilized on hydrophobic surfaces, and Novozym 435 (CALB with acrylic immobilization), which is immobilized by macroporous acrylic polymeric resin [24] with recombinant Aspergillus niger, thereby enabling a process with high epoxide yields [25]. Vlcek and Petrovic [16] established a procedure for soybean oil epoxidation catalyzed by Novozym 435 which overcomes the double bond conversion by 90%.

Novozym 435 is applied in epoxidations due to its high enzymatic activity. The most investigated oils for epoxidation with Novozym are soybean [22,23,24,25,26], linseed [27], corn [28], and sunflower [29]; the literature also reports oleic acid [30]. This enzyme efficiency during the epoxidation process of vegetable oils is due to the presence of carboxylic acids present in the oils, which transport oxygen [25,26,27,28,29,30,31], allowing the peracid produced from the aqueous phase interaction (between hydrogen peroxide and water) to react in the phase with the enzyme [32].

In the present work, the enzymatic epoxidation of castor oil was investigated using systems with a lipase catalyst immobilized in acrylic resin (Novozym 435) and lipase only with immobilization in Candida antarctica with the purpose of analyzing the behaviour of the enzyme when added to systems with and without toluene. The conversion rates, epoxidation, and selectivity were compared with synthetic reactions based on quantitative evaluations performed using ^1^H-NMR applying distinct reaction times. The epoxidized castor oil turned out to be an alternative for other oils from the food industry when producing epoxy resins. Additionally, it was investigated as an appropriate system for the enzyme lipase without acrylic immobilization in order to enable its use in epoxidation reactions, replacing Novozym.

## 2. Experimental

### 2.1. Materials

Castor oil was purchased from Dinâmica Química (Indaiatuba, Brazil). Hydrogen peroxide 35% was purchased from NEON (Suzano, Brazil). Acetic acid, sulfuric acid, diethyl ether, sodium sulfate, benzene, immobilized lipase enzyme from Candida antarctica, Amberlite IRC120H ion exchange resin, chloroform-d, and Novozym’s acrylic-immobilized lipase enzyme were purchased from Sigma–Aldrich (St. Louis, MI, USA). Toluene was supplied by Honeywell (Charlotte, NC, USA).

### 2.2. Methodology

#### 2.2.1. Synthetic Epoxidations

The synthetic epoxidations of castor oil were performed based on the adapted methodology of Sinadinovic et al. [12] and Saremi et al. [7]. The reactions were carried out for 24 h in a two-neck round-bottom flask coupled to a reflux condenser with a JAD model SP-500 submersible pump (Beijing, China) and an additional funnel in a water bath at 50 °C with magnetic stirring at 240 rpm using a Corning Incorporated PC420D magnetic plate (Corning, New York, NY, USA). Initially, the reaction was performed using 1.25 g (5% of the weight) of Amberlite IRC120H ion exchange resin, 25 g of castor oil, 28.5 mL of benzene, and 22.5 mL of acetic acid. Likewise, in the reaction without the acrylic resin, the same amounts of castor oil and hydrogen peroxide were added to 8 mL of formic acid (without benzene and acetic acid addition).

After homogenization of the above-mentioned components, hydrogen peroxide was added drop by drop. At the end of the reaction, the organic phase was extracted with ethyl ether, washed with distilled water to remove acid residues, and dried with sodium sulfate.

#### 2.2.2. Chemoenzymatic Epoxidations

In order to compare the enzymatic behavior of lipase with acrylic immobilization and lipase with immobilization in Candida antarctica without acrylic immobilization, the protocols reported by Vlcek et al. [16] and Zhang et al. [33] were applied with subtle modifications. The reactions lasted 24 and 6 h at temperatures of 45 and 110 °C, respectively.

The 24 h enzymatic epoxidation reactions were carried out in a round-bottomed, two-necked flask coupled to a reflux condenser with a JAD model SP-500 submersible pump (Beijing, China) and an additional funnel in a water bath at 110 °C at 200 rpm with magnetic stirring using a Corning Incorporated PC420D magnetic plate (Corning, New York, NY, USA). For the reaction with acrylic immobilization, 4 g of castor oil and 10% (0.4 g) of Novozym 435 (Novozym 435 features an acrylic immobilization that enables its stability in the reaction medium and has longer reaction times for complete epoxidation in mild systems, enabling its reuse for new enzymatic reactions) were added. For the reaction without acrylic immobilization, 5 g of castor oil and 10% (0.5 g) lipase enzyme in Candida antarctica (as it presents only one immobilization in Candida Antarctica, its interaction with the reaction medium is greater, making it reactive, so it does not have the characteristic of being reusable) and 12.68 g of toluene were added. The dropwise addition of 2.33 mL of hydrogen peroxide marked the zero point (in time). At the end of the reaction, the enzyme was separated from the reaction mixture by vacuum filtration and washed with toluene for reuse. Distilled water was added to the reaction mixture, which was transferred to a separation funnel, and the organic phase was dried with anhydrous sodium sulfate after separation.

The reactions with enzyme without acrylic immobilization were also carried out using a round double-neck flask coupled to a reflux condenser and dropping funnel immersed in a water bath at 45 °C for 6 h under magnetic stirring at 200 rpm, using 5 g of castor oil, 4% (0.2 g) of the weight of Candida antarctica lipase enzyme without acrylic immobilization, 2.33 mL of hydrogen peroxide, and 6 mL of toluene (Table 1). In the end, the reaction mixtures were vacuum filtered to remove the enzyme, which was washed with toluene for later use. The reaction was stabilized with distilled water; the organic phase was separated and dried with sodium sulfate.

#### 2.2.3. Fourier Transform Infrared Spectroscopy (FTIR)

FTIR analysis was performed with castor oil and epoxidation reaction specimens without any type of solvent. The tests were conducted using a Shimadzu model IR Prestige-21 spectrophotometer (Tokyo, Japan). The coupled attenuated total reflectance accessory was applied, using the 4000–600 cm^−1^ region as the analysis condition. Resolution: 4 cm^−1^; number of accumulations: 20; mode: transmittance, using the SigmaPlot 12.3 software from Systat Software Inc. (San Jose, CA, USA) for spectral analysis.

The transmittance conversion into absorption was computed using the modified Beer–Lambert law [34], according to Equation (1):

(1) AU=2−log%T

where AU is the absorbance, −log is the natural logarithm and %T is the wave transmittance percentage.

#### 2.2.4. Nuclear Magnetic Resonance (^1^H-NMR)

For the analysis of ^1^H-NMR, specimens with 30 mg (castor oil and reaction products) were dissolved in 0.6 mL of chloroform-d. The analyses were carried out using a Mercury 500 spectrometer from Varian Medical Systems (Palo alto, CA, USA) operating at 400 and 500 MHz for ^1^H-NMR, and data analyses were performed using the MestreNova software.

Using the normalization factor (Equation (2), which considers the relative area of the hydrogen signals of the glycerol methylene groups (the integral of these signals), the initial and final number of double bonds (Equation (3), as well as the conversion rates (Equation (4), degree of epoxidation (Equation (5), and degree of selectivity (Equation (6), were computed [35,36]

(2) $$NF=\frac{B}{4}$$

(3) $$ND=\frac{A-NF}{2NF}$$

(4) $$Conversion\left(\%\right)=\left[\frac{{ND}_{i}-{ND}_{f}}{{ND}_{i}}\right]\times 100$$

(5) $$Epoxidation\left(\%\right)=\left[\frac{\frac{C}{2}}{NF\times {ND}_{i}}\right]\times 100$$

(6) $$Selectivity\left(\%\right)=\left[\frac{Epoxidation\left(\%\right)}{Conversion\left(\%\right)}\right]\times 100$$

where NF is the normalization factor, B is the area of the glycerol group’s hydrogen signal, ND is the number of double bonds, A is the signal area of the hydrogens at unsaturated carbon atoms, and C is the signal area attributed to the epoxide ring hydrogens.

## 3. Results and Discussion

### 3.1. Infrared Spectroscopy Analysis of the Synthetic Reactions

**Figure 1:**
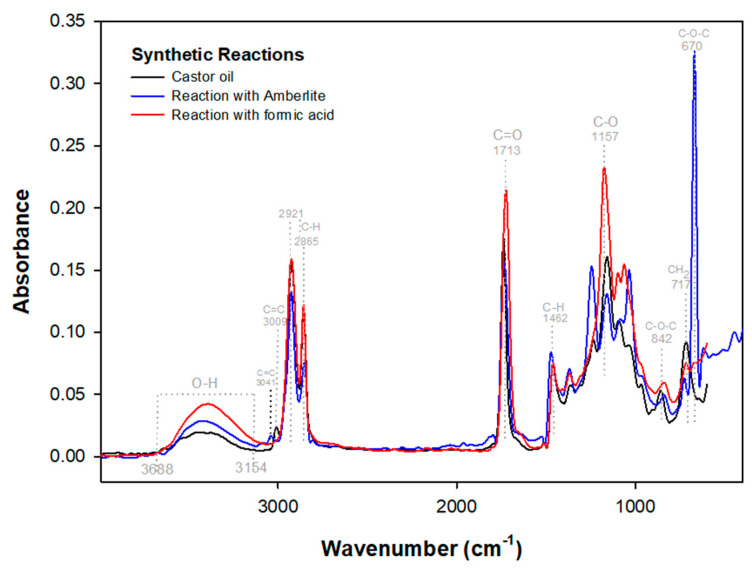
FTIR spectra of castor oil and the synthetic reactions’ products.

**Figure 2:**
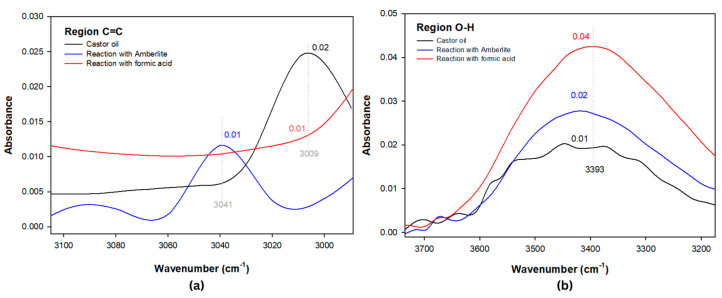
Spectra zoom for the hydroxyl (**a**) and unsaturation (**b**) regions of the synthetic reactions.

**Figure 3:**
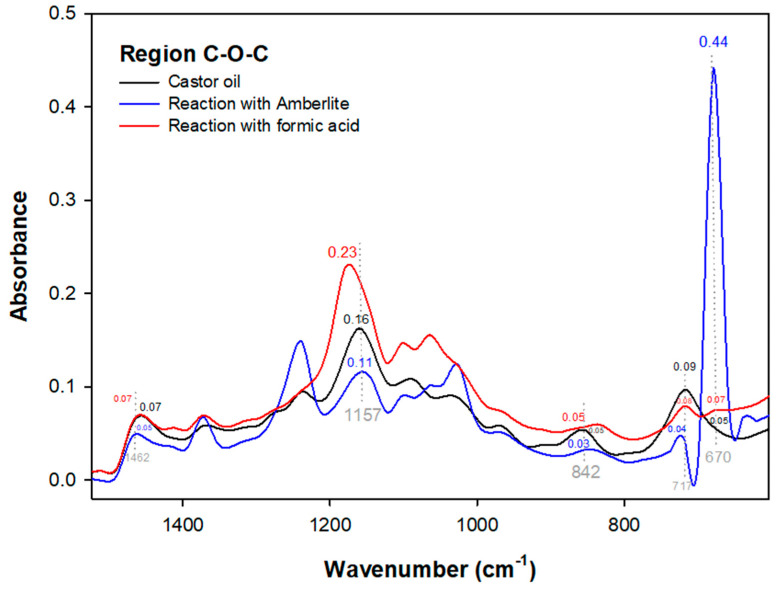
FTIR spectra for the epoxy region of the synthetic reactions.

Figure 1 shows the collected spectra for castor oil after its reaction with Amberlite and formic acid. The synthetic reaction with Amberlite IR-120 displayed a band shift from 3009 cm^−1^ to 3041 cm^−1^, referring to the predominant unsaturated ricinoleic acid (Figure 2a), corroborating changes in the epoxide area between 1462 and 594 cm^−1^ (Figure 3) where unfolding and C-H stretching (1462 cm^−1^), C-O (1157 cm^−1^), and CH_2_ (717 cm^−1^) were observed. In addition, the peak at 670 cm^−1^ emphasizes the appearance of an oxirane ring with hydrogen angular deformation [37].

In Figure 2, the unsaturation region (a) referring to the Amberlite reaction points to a peak displacement at 3041 cm^−1^. However, in the reaction with formic acid, the absorption peak at 3009 cm^−1^ of the C=C bond is verified, indicating that the difference in events is related to the unsaturation conversion into an oxirane ring, while in the reaction with Amberlite, the appearance of the peak at 670 cm^−1^ promotes the displacements and absorptions in the C-H bonds of the epoxy region (1462–650 cm^−1^) and of the peaks at 2921 and 2865 cm^−1^, promoting the unsaturation displacement of the synthetic reaction with Amberlite. In the reaction with formic acid, despite not promoting the appearance of a new peak characteristic of the epoxy ring, the conversion of the peak at 3009 cm^−1^ is related to hydroxyl stretching, which is verified at 3393 cm^−1^ (Figure 2b), and C-O absorption (1157 cm^−1^), indicating modification in the epoxy group.

The reaction with formic acid presented similar changes in the epoxy region (Figure 3) when compared to the synthetic epoxidation with Amberlite, albeit with less intensity. This is because intensities such as the band at 670 cm^−1^ of the Amberlite reaction indicate epoxidation events in the material and are thus needed to detect castor oil epoxidation events. These events were not verified for the reaction in the formic acid medium due to recurrent phenomena involving double bond conversion and the presence of H_2_O in the hydroxyl region (3688–3154) that promote a peak elongation at 3393 cm^−1^ (Figure 2b), and allow the epoxy group to open [7], which in turn indicates the oxirane ring modification.

In the epoxy group region (Figure 3), the band at 842 cm^−1^ stands out. This is verified in the castor oil spectrum which has a peak absorbance at 860 cm^−1^. A similar result was reported by Bock et al. [38], where the same behavior was detected in a band at 814 cm^−1^ for the enzymatic reaction of castor methyl ester, a behavior also analyzed in the enzymatic reactions previously mentioned in this study, corroborating that behaviors in existing peaks in the epoxy group indicate the presence of oxirane.

### 3.2. Infrared Spectroscopy Analysis of Enzymatic Reactions

**Figure 4:**
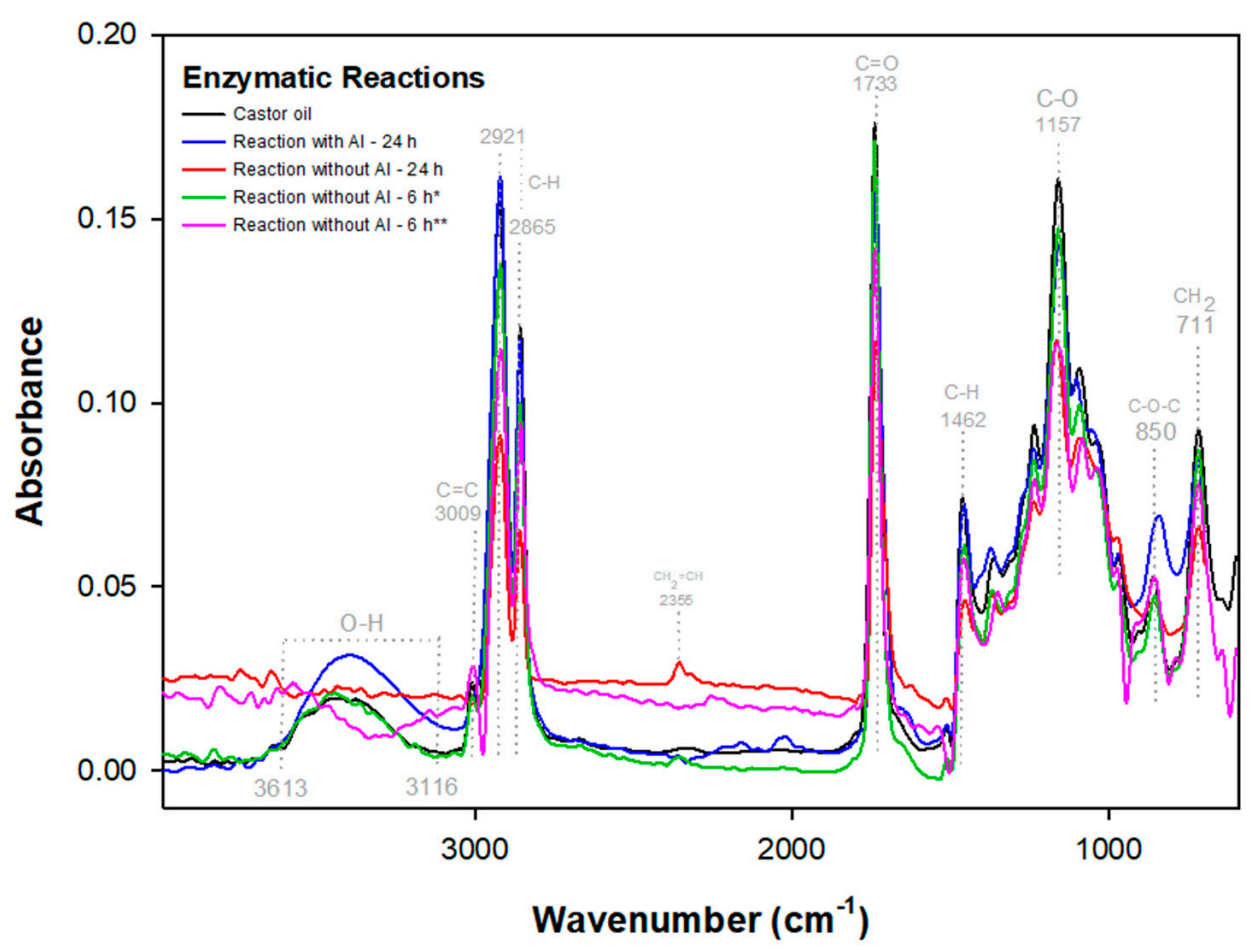
FTIR spectra for the enzymatic epoxidations. * With toluene; ** without toluene.

To examine the effects of enzymatic epoxidation reactions, FTIR analyses focused on the main functional group bands present in the hydroxyl regions (O-H) at 3613–3116 cm^−1^, predominant double bond (C =C) at 3009 cm^−1^, and the epoxy region (C-O-C) 1462–600 cm^−1^ were conducted. In Figure 4, the enzymatic epoxidations with acrylic immobilization (with AI) and without acrylic immobilization (without AI) are displayed for the reaction times of 6 and 24 h (in the case of epoxidation of castor oil with a concentration of 88% ricinoleic acid, the reaction does not necessarily come from the isolation of a specific fatty acid, thus the modifications of the peaks at 3397, 3009, and 850 cm^-1^ already existing in the FTIR spectrum of the neat oil are analyzed.) [39].

Investigating the hydroxyl group present in castor oil (Figure 4) using the Beer–Lambert law, an absorbance of 0.02 AU (97%T) in the bands between 3613 and 3116 cm^−1^ for the reaction with IA, referring to the hydroxyl group present in ricinoleic acid, is observed. Consequently, an increase in water in its structure, an expected event in vegetable oil epoxidation, suggests a possible occurrence of oxirane ring opening. This corroborates the system proposed by Lewandowski et al. [40] where, during the triglycerols epoxidation mechanism and fatty acid alkyl esters, the occurrence of perhydrolysis in the enzymatic epoxidation reaction with lipase, characterized by the appearance of H_2_O, a by-product of H_2_O_2_, results in the removal of oxygen during oxirane ring production in the final system.

**Figure 5:**
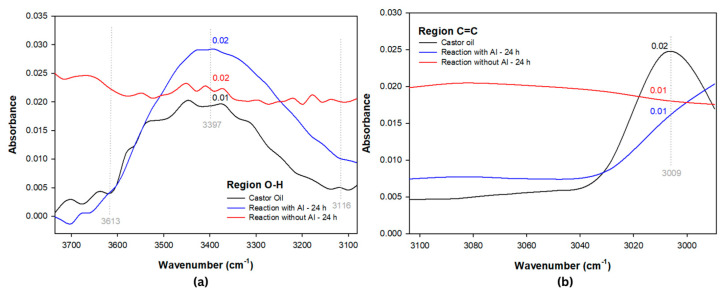
Zoom of FTIR spectra of hydroxyl (**a**) and unsaturation (**b**) regions for the enzymatic reactions at 24 h.

In the epoxidation without AI (24 h), Figure 5a, the hydroxyl group, which has a transmittance of 94% (3613–3116 cm^−1^), lower than that of the neat castor oil (99%), stands out. In addition, the absorbance remained constant at 0.02 AU (Figure 5), as opposed to neat castor oil, where the beginning and end absorbance values were 0.00 AU (3982 cm^−1^), with a stretch of 0.01 AU occurring only at 3403 cm^−1^, thus indicating a loss of H_2_O due to a dehydration event since the reaction occurred above the water boiling point, at 110 °C, verifying a possible appearance of the vinyl group at 2355 cm^−1^ (Figure 4) [41].

**Figure 6:**
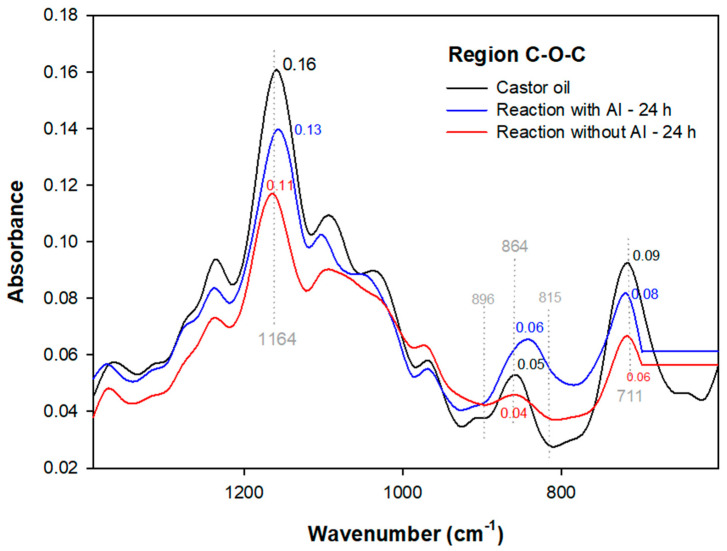
FTIR spectra of the epoxy region for the enzymatic reactions at 24 h.

In the bands at 3009 cm^−1^ in the reaction with IA (Figure 5b), the peak referring to the double bond disappeared, promoting a stretching at 864 cm^−1^ (Figure 6) and resulting in an absorbance of 0.06 AU, indicating the unsaturation conversion into an epoxy group, which was subsequently detected in the ^1^H-NMR analysis [36].

In the enzymatic reaction without AI (24 h), the characteristic peak of the ricinoleic acid double bond at 3009 cm^−1^ (Figure 5b) was removed by the peroxide reaction with lipase, promoting folding events (Figure 6) of the glycerol and triacylglycerol group bonds (1164, 864, and 711), as verified by Hernandez et al. [36] who, in a castor oil catalytic epoxidation system, identified axial flexion and bending of the oxirane ring at 870 cm^−1^ and 846 cm^−1^ related to this group’s polarization.

**Figure 7:**
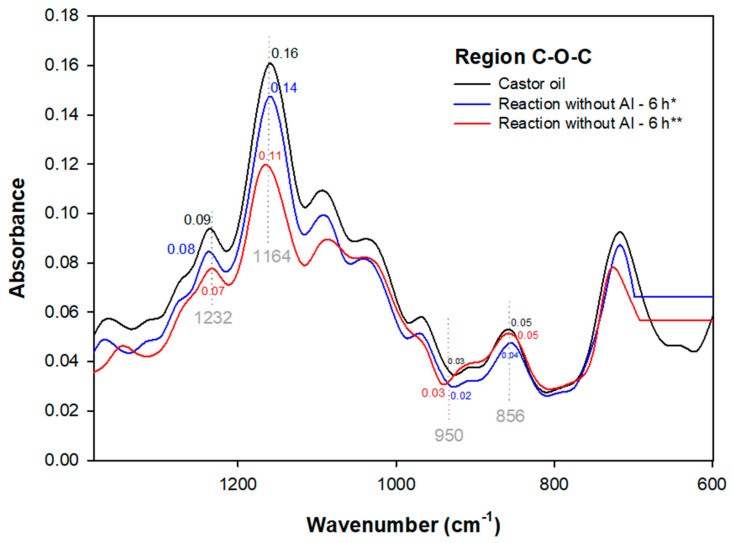
FTIR spectra of the epoxy region for the 6 h enzymatic reactions. * With toluene; ** without toluene.

In Figure 7, two systems of enzymatic epoxidation without AI are highlighted, with temperature ranging from 40 to 45 °C and with or without toluene addition.

**Figure 8:**
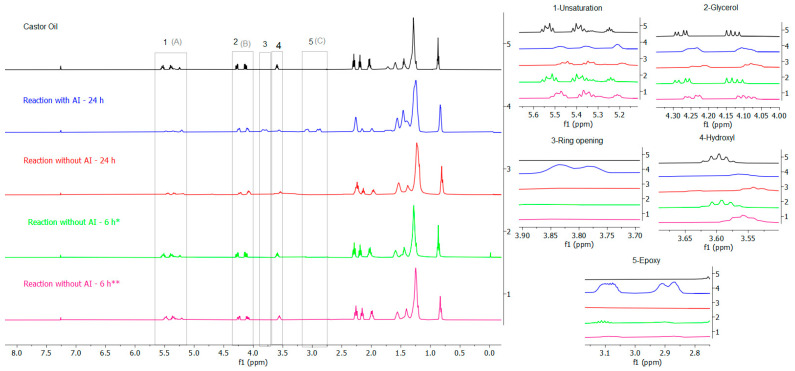
^1^H-NMR spectra for the enzymatic reactions. * With toluene; ** without toluene.

In the non-toluene reaction during 6 h performed at 45 °C, a deformation of the vinyl group unsaturation was verified [42]. In addition, the peak stretching at 950 cm^−1^ (Figure 8), characteristic of the oxirane ring, was verified by a change in the absorbance (Figure 4) from 0.03 AU of castor oil to 0.01 AU, corroborating the castor oil epoxidation due to the peak displacement of the C=C bond (Figure 4), which is related to deformation of the vinyl group plane unsaturation.

There is also the hydroxyl modification at 3613–3116 cm^−1^ (Figure 4), indicated by a peak with an absorbance of 0.00 AU (Figure 4), where in castor oil it is 0.01 AU, verifying the peroxide H_2_O loss.

In the FTIR of Figure 4, the 6 h at 40 °C enzymatic reaction with toluene did not display changes in the unsaturation regions (3009 cm^−1^) and hydroxyl (3403 cm^−1^). Considering this result, hydrogen peroxide decomposition and enzyme inactivity impair the unsaturation conversion to an oxirane ring due to the non-acrylic immobilization of the enzyme. However, there was absorbance in peaks 1232 (0.08 AU), 1164 (0.14 AU), and 856 cm^−1^ (0.04 AU) referring to triglycerides (Figure 7). As a result of the oxirane ring deformation and the polarization of its C-O bond, the formation of a three-dimensional polymeric network structure is possible [1,43,44].

Considering the effects of 24 and 6 h as well as the acrylic immobilization and immobilization in Candida antarctica of the tested enzymes, it was verified that the lipase without acrylic immobilization (without AI) in the 24 h reaction (Figure 5a) displayed a stretching of the hydroxyl region (3397 cm^−1^) due to greater contact between the enzyme without AI and the peracid, promoting H_2_O appearance. On the other hand, the 6 h reaction (without toluene), Figure 4, promoted O-H (3420 cm^−1^) group loss, indicating that the enzyme without AI reaches castor oil dehydration in a shorter time.

The 6 h enzymatic reaction without AI using toluene as an organic solvent provided a decrease in the hydroxyl absorption (0.01 AU) (Figure 4) compared to the 24 and 6 h reactions mentioned above. However, a comparative analysis with the 24 h enzymatic reaction with acrylic immobilization (with AI) revealed that its hydroxyl promotes the possibility of dehydration better than the oxirane ring opening. This fact was confirmed by ^1^H-NMR analyses of the AI reaction where an oxirane ring opening peak was detected, corroborating the absorption of 0.02 AU in Figure 5a. This indicates that the interference of hydroxyl conversion during enzymatic reactions is due to the use of toluene as an organic solvent, given the non-addition of this solvent in the enzymatic reaction with AI (24 h).

Regarding these data, it was also verified that the enzyme lipase without acrylic immobilization did not promote the unsaturated peak (3009 cm^−1^) removal in the 6 h reactions, indicating that the enzyme without AI promoted the hydroxyl dehydration at that time but removed the unsaturation of castor oil in 24 h systems, a fact that can also be related to the applied temperatures in the reactions of 24 (110 °C) and 6 h (45 °C). This is because in ranges below 50 °C, the enzyme without AI promotes changes in the hydroxyl group; in contrast, at high temperatures, the enzyme with AI modifies C=C groups and has low reactivity, unlike lipase without AI that, since it does not have acrylic immobilization, becomes reactive in the system with peracid.

### 3.3. Quantitative Analysis by ^1^H-NMR of Epoxidation Reactions

Acquired data collected through FTIR for the enzymatic and non-enzymatic reactions were corroborated using NMR. As can be seen in Figure 8, the region of epoxidation site unsaturation (5.5 and 5.0 ppm) for the enzymatic reaction with IA (Figure S3) indicated 50% conversion (Table 2), and in the enzymatic epoxidation without AI (Figure S4), 45% of the oil was converted. Although the two reactions showed similar conversion rates, there was a difference in the selectivity. The reaction with AI showed 1% of oxirane conversion and 2% of selectivity while the reaction without AI showed 10% of oxirane conversion and 22% of selectivity; these data are presented in Table 2, with both reactions proceeding in 24 h.

In a study of enzymatic epoxidation (with acrylic immobilization) with soybean oil, Zhang et al. [33] obtained conversions between 84 and 99% at 35 °C, where toluene addition and temperature can be considered as different parameters. It was stated that at 50 °C, reactions with toluene provided an epoxidation of 80%. In the present work, the results showed that the reaction without AI had an increase in epoxidation of 9% compared to the reaction with AI, showing that despite the high temperatures, the enzyme without acrylic immobilization performed a lower amount of unsaturation conversion in 24 h of reaction; nevertheless, its epoxidation with toluene is effective.

However, in the two 24 h enzymatic reactions, the signals at 4.4 and 4.0 ppm referring to the glycerol hydrogens, as well as the hydroxyl hydrogen at 3.5 ppm, changed. Furthermore, in the epoxidation reaction with AI (24 h), there was a signal appearance at 3.9 and 3.7 ppm, attributed to hydrogens resulting from the epoxide ring opening [45] highlighted by the presence of a di-epoxide in the region of 3.0 ppm. Hernandez et al. [36] acquired a similar result with castor oil, where a by-product that was attributed to epoxidized castor oil-polyol was verified.

Unlike the 24 h enzymatic reactions, the 6 h enzymatic reaction without AI and without toluene (Figure 8) did not show chemical displacement or ^1^H-NMR (Figure S6) signs for the epoxide ring production, only decreases in the intensity, confirming the acquired results in the FTIR section. However, the hydroxyl hydrogen signal disappearance due to the folding event of the glycerol groups of the triacylglycerols and slight changes in the signals of the CH_2_ group between 2.3 and 1.1 ppm during the enzymatic reaction without AI (24 h) suggested 50% of conversion and 2% of selectivity (Table 2), matching the enzymatic reaction with AI (24 h), in addition to an epoxidation of 28%, an increase of 18% compared to epoxidation without AI (24 h).

Nevertheless, in the enzymatic reaction without AI (6 h) with toluene as solvent (Figure S5), the same behavior as the reaction without solvent was verified with the unsaturation peaks region (5.6 to 5.2 ppm), glycerol (4.3 to 4.0 ppm), and hydroxyl (3.6 to 3.5 ppm) showing only decreases in intensity, providing 50% of C=C bond conversion. In addition, hydroxyl chemical shifts initiated a triplet appearance with lower intensity at 3.1 ppm, contributing to a conversation rate of 25% and reaching a selectivity of 50%, 48% greater than the reaction without AI (6 h) and without toluene. Therefore, indicating the same conversion rate and an approximate epoxidation, the enzymatic reactions without acrylic immobilization differ in their quantitative results by the reaction time and not necessarily by the added solvent (toluene), as it does not provide considerable differences in the unsaturation conversion as well as in the oxirane appearance.

It should be noted that the difference in enzymatic epoxidation rates at 24 and 6 h are significant due to the conditions of the enzymes used, keeping in mind that, in the 6 h reactions, using the enzyme without acrylic immobilization allows for an interaction with the peracid formed in the reaction system, allowing results such as 25% in the reaction without AI (6 h) and without toluene; however, this non-immobilization promotes the deactivation of the enzyme during longer reactions, as analyzed in the reaction without AI for 24 h. In addition, the non-use of the organic solvent (toluene) in systems with AI and without AI also resulted in a decrease in epoxidation rates of 1 and 10%, respectively.

**Figure 9:**
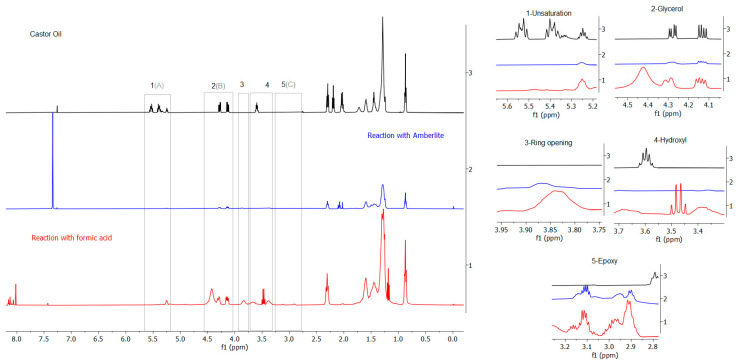
^1^H-NMR spectra for the synthetic reactions.

Figure 9 shows the synthetic reactions with the solid catalyst Amberlite IR-120 and with formic acid, respectively, both for 24 h. ^1^H-NMR spectra for the reaction with the acrylic resin displayed suppression of unsaturation signals from 5.6 to 5.2 ppm and CH_2_ of triacylglycerol from 2.4 to 1.0 ppm (Figure S1), rendering a 63% of conversion for the C=C bond (Table 2). The spectra also showed that glycerol and hydroxyl groups disappear, suggesting possible epoxide formation followed by its opening, resulting in 48% epoxidation and 76% selectivity.

Sinadinovic-Fiser et al. [12] reached 68% of conversion for castor oil epoxidation with 10% by weight of Amberlite in addition to 46.22% epoxy yield and 0.68 selectivity in a 25 h reaction. When adding the same percentage (%) of the added weight in this work (5%) with a time of 10 h, the authors acquired 83.2% of conversion, 70.75% of yield, and 0.85 selectivity. Therefore, parameters such as higher catalyst concentration and shorter reaction times are of fundamental importance for the epoxidation reaction of castor oil with Amberlite to have a high conversion rate.

The synthetic reaction with formic acid (Figure 9) also showed signs of unsaturation degradation, indicating 96% of conversion; however, it obtained 6% of epoxy yield and selectivity (Table 2) due to chemical shifts in the glycerol (4.5 to 4.1 ppm) and hydroxyl (3.7 to 3.4 ppm) regions, showing only traces of the epoxy group. According to Saremi et al. [7], as a result of ring opening, the occurrence of epoxy opening produces hydroxyl functional groups as a side reaction, elucidating the identified chemical shifts. Furthermore, Luca et al. [45], when identifying the characteristic sign (Figure S2) appearance of the ring opening in a 78% of conversion, stated that the addition of formic acid to epoxidized castor oil provides the oxirane ring cleavage.

## 4. Main Gains

In the enzymatic epoxidations, it was verified that for the 24 h systems with and without acrylic immobilization, there was OH group absorption (0.02 and 0.06 AU) and C=C (0.01 AU) by the peracid reaction with the lipase, allowing only a 5% difference in the conversion rate between the two reactions. However, considering a rate of 45% for enzymatic epoxidation without acrylic immobilization (24 h), the possible vinyl group appearance promoted an increase of 10 and 20% in the epoxidation and selectivity rates, respectively, a fact established by the appearance of a product derived from the ring opening that was indicated in the ^1^H-NMR analysis, resulting in a reaction rate decrease in the reaction with acrylic immobilization.

The enzymatic epoxidations without acrylic immobilization and reaction time of 6 h (with and without toluene), as well as the synthetic epoxidations, indicated less interference in the OH and C=C groups, with the exception of the enzymatic reaction without AI (6 h without toluene), which displayed a hydroxyl peak absorption, but without the unsaturation removal, and the synthetic reaction with formic acid that presented a peak elongation in the hydroxyl (3391 cm^−1^) and unsaturation loss (3009 cm^−1^), thus promoting greater unfolding and stretching events in the C-H, C-O, C=O, and CH_3_ groups. This increased the conversion rates from 50 to 96% for enzymatic reactions without AI (6 h) and synthetic ones with and without solid catalyst and resulted in a decrease of 42 % variance between the epoxidation rates of the enzymatic reaction without AI (6 h) without toluene and the synthetic reaction with Amberlite.

## 5. Conclusions

In this work, castor oil was epoxidized using lipase enzymes with and without acrylic immobilization and in synthetic systems with the solid catalyst Amberlite and performic acid. Its conversion rates, epoxidation, and selectivity were analyzed using NMR and corroborated through FTIR.

Based on the acquired data, the enzymatic epoxidation systems performed with the lipase enzyme without acrylic immobilization resulted in modifications of castor oil hydroxyl peaks, as well as in the double bond; the time and added enzyme effects were evaluated, which resulted in the conversion of 50 and 45% of the unsaturation peak (3009 cm^−1^) for the 24 h reactions with and without acrylic immobilization, respectively, in addition to alterations in the hydroxyl band (3397 and 3420 cm^−1^), conducting to oxirane ring opening (3.85–3.75 ppm) in the epoxidation with AI; the epoxidation rate and selectivity were 1 and 2%, respectively.

However, for the reaction without AI (24 h), the 45% conversion resulted in a possible vinyl group (2355 cm^−1^), a recurrent product of the secondary reaction of hydroxyl dehydration, from the reactive contact of the catalyst without acrylic immobilization with peracid, promoting absorption of 0.02 AU trough H_2_O removal due to the reaction time of 24 h, resulting in epoxidation and selectivity rates of 10 and 22%, respectively.

The enzymatic epoxidation without acrylic immobilization with a reaction time of 6 h and the synthetic epoxidation of 24 h showed better conversion rates. Except for the reaction with formic acid, where the conversion was 96%, the epoxidation and selectivity of 6% indicates that castor oil performs conversion of almost 100% in systems without solid catalysts. Still, its addition is necessary for epoxidation to take place. In addition, organic solvents and reaction time interfere directly with the conversion rates of epoxidation with the enzyme without AI promoting epoxidation of 25 and 28% in 6 h of reaction with and without organic solvent, respectively, and 31% with Amberlite and benzene.

## Figures and Tables

**Table 1:** Information on the enzymatic reactions performed.

Reactions	Time (h)	Castor Oil (g)	Enzyme (% by Weight)	H_2_O_2_ (mL)	Toluene (mL)
With AI *	24	4	10	3.45	0
Without AI **	24	5	10	2.33	12.68
Without AI	6	5	4	2.33	6
Without AI	6	5	4	2.33	0

* With acrylic immobilization; ** without acrylic immobilization.

**Table 2:** Quantitative data acquired using ^1^H-NMR for the performed reactions.

Reactions	Time (h)	Conversion (%)	Epoxidation (%)	Selectivity (%)
With AI	24	50	1	2
Without AI	24	45	10	22
Without AI *	6	50	25	50
Without AI **	6	50	28	2
With Amberlite	24	63	48	76
With Formic Acid	24	96	6	6

* With Toluene; ** without Toluene.

## Data Availability

Data will be made available on request.

## Supplementary Materials

The following supporting information can be downloaded at: https://www.mdpi.com/article/10.3390/polym15112477/s1, Figure S1: ^1^H-NMR of the synthetic epoxidation reaction with Amberlite; Figure S2: ^1^H-NMR of the synthetic epoxidation reaction with formic acid; Figure S3: ^1^H-NMR from the 24 h enzymatic epoxidation reaction with AI; Figure S4: ^1^H-NMR from the 24 h enzymatic epoxidation reaction without AI; Figure S5: ^1^H-NMR from 6 h enzymatic epoxidation reaction without AI with toluene; Figure S6: ^1^H-NMR from 6 h enzymatic epoxidation reaction without AI without toluene.
