# Supplementary material for: Enzymatic and Synthetic Routes of Castor Oil Epoxidation
Source: Polymers (Basel). 2023 May 27;15(11):2477. doi: 10.3390/polym15112477 (PMC10255699; doi:10.3390/polym15112477)
Supplement: Supplementary file 1 [file polymers-15-02477-s001.zip › polymers-2380935-supplementary.pdf]

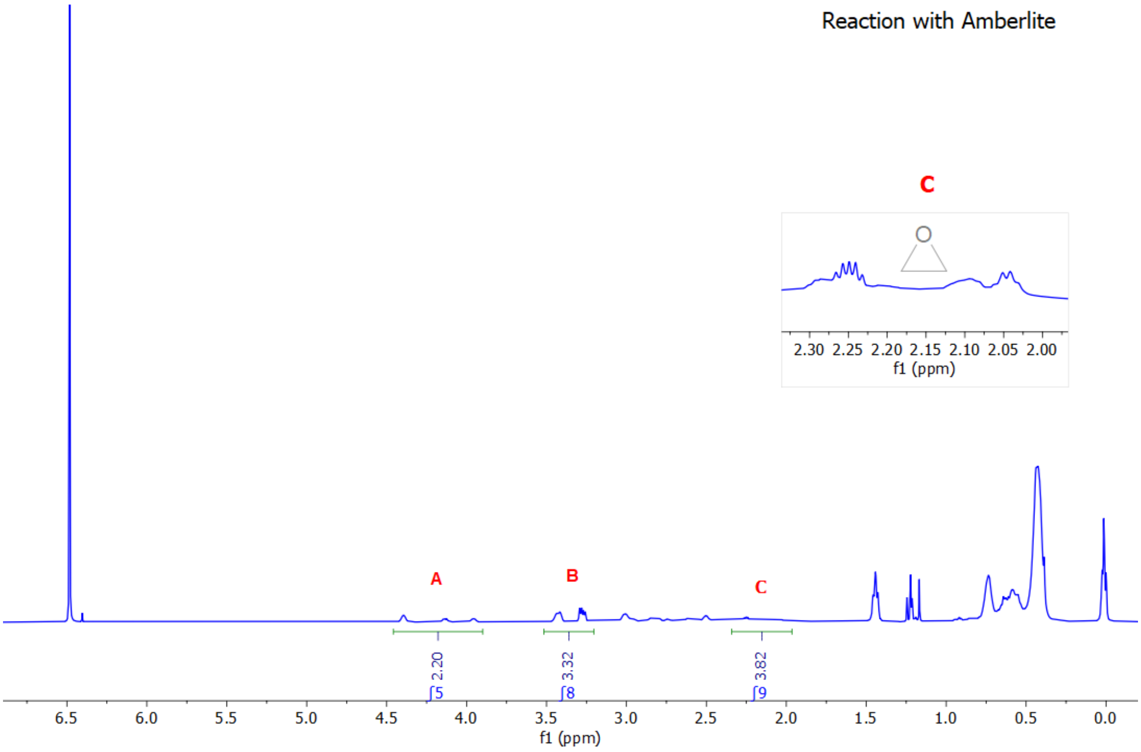

Figure S1: ^{1}H-NMR of the synthetic epoxidation reaction with Amberlite.

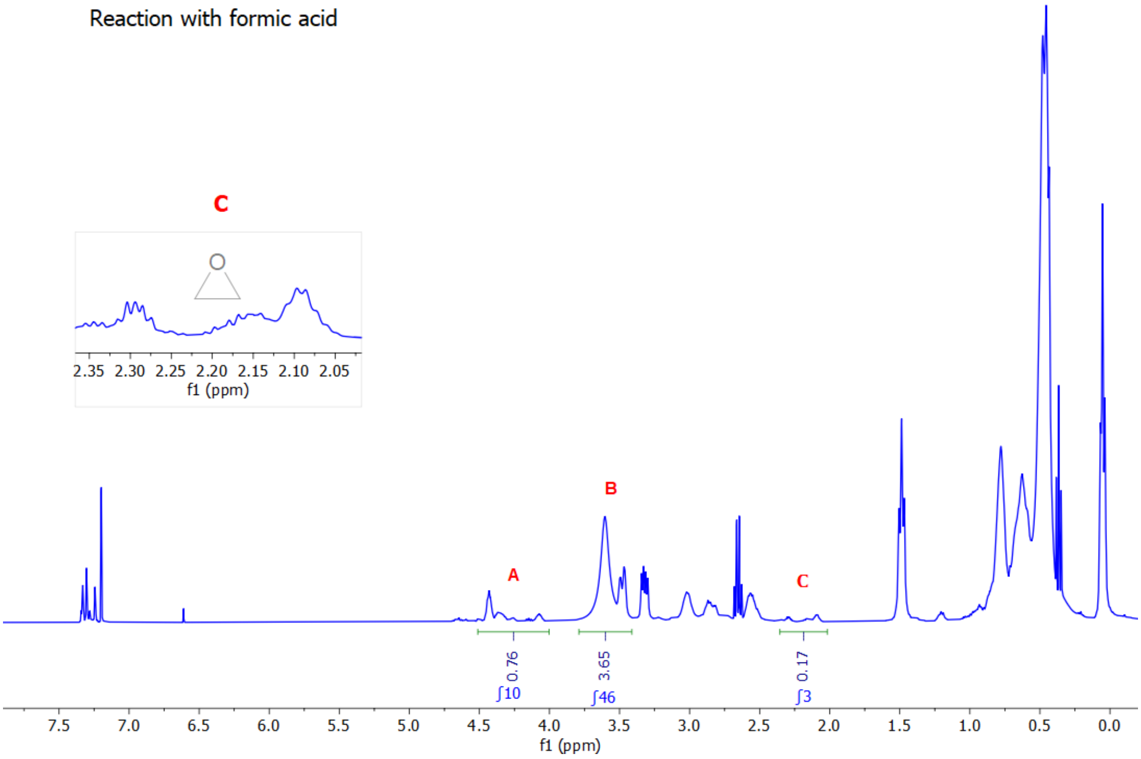

Figure S2: ^{1}H-NMR of the synthetic epoxidation reaction with formic acid.

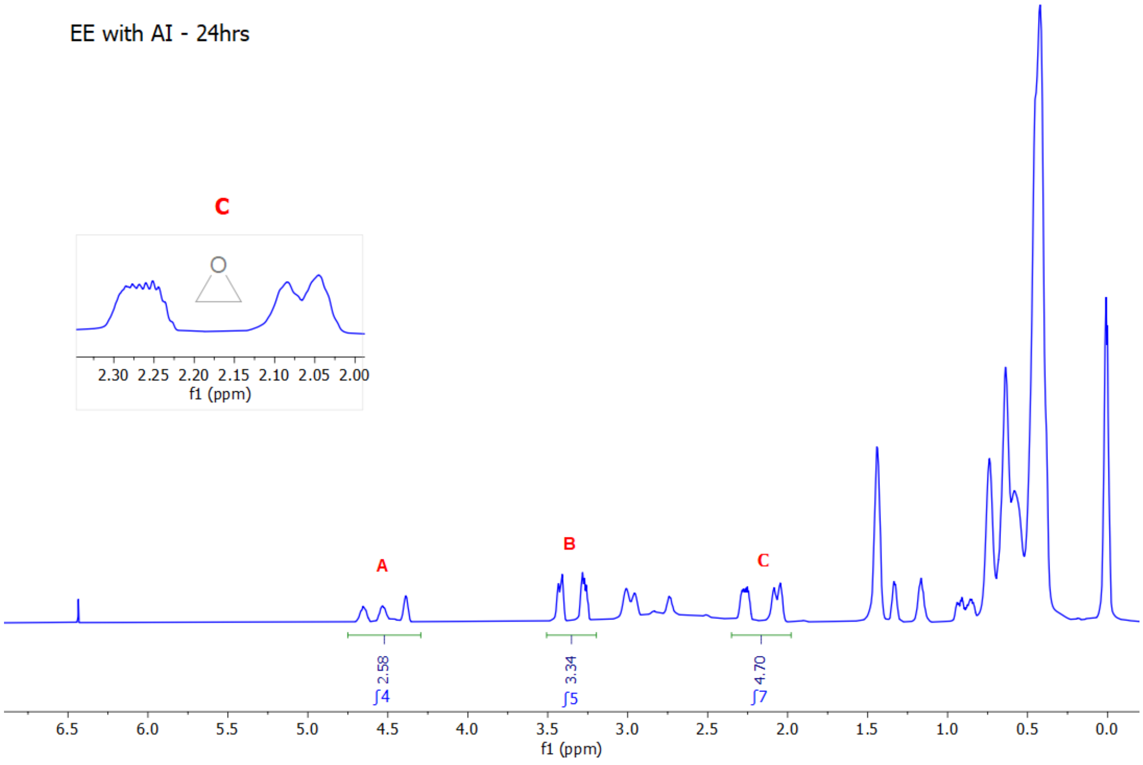

Figure S3: ^{1}H-NMR from the 24 hr enzymatic epoxidation reaction with AI.

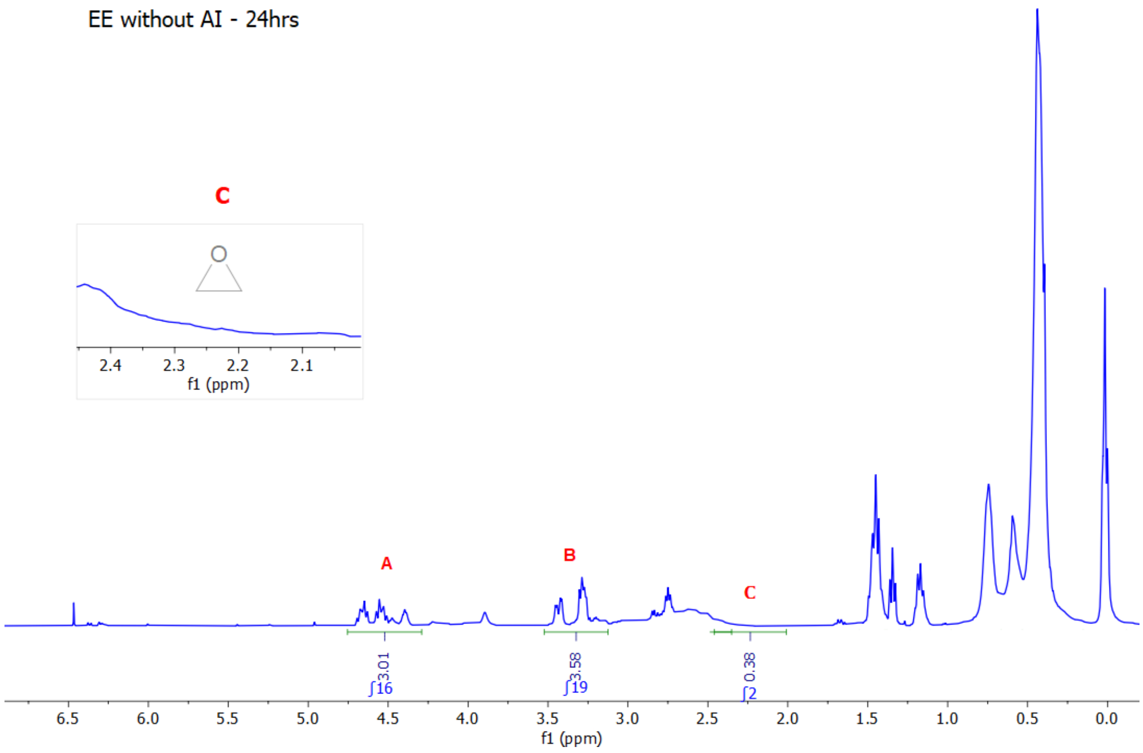

Figure S4: ^{1}H-NMR from the 24 hr enzymatic epoxidation reaction without AI.

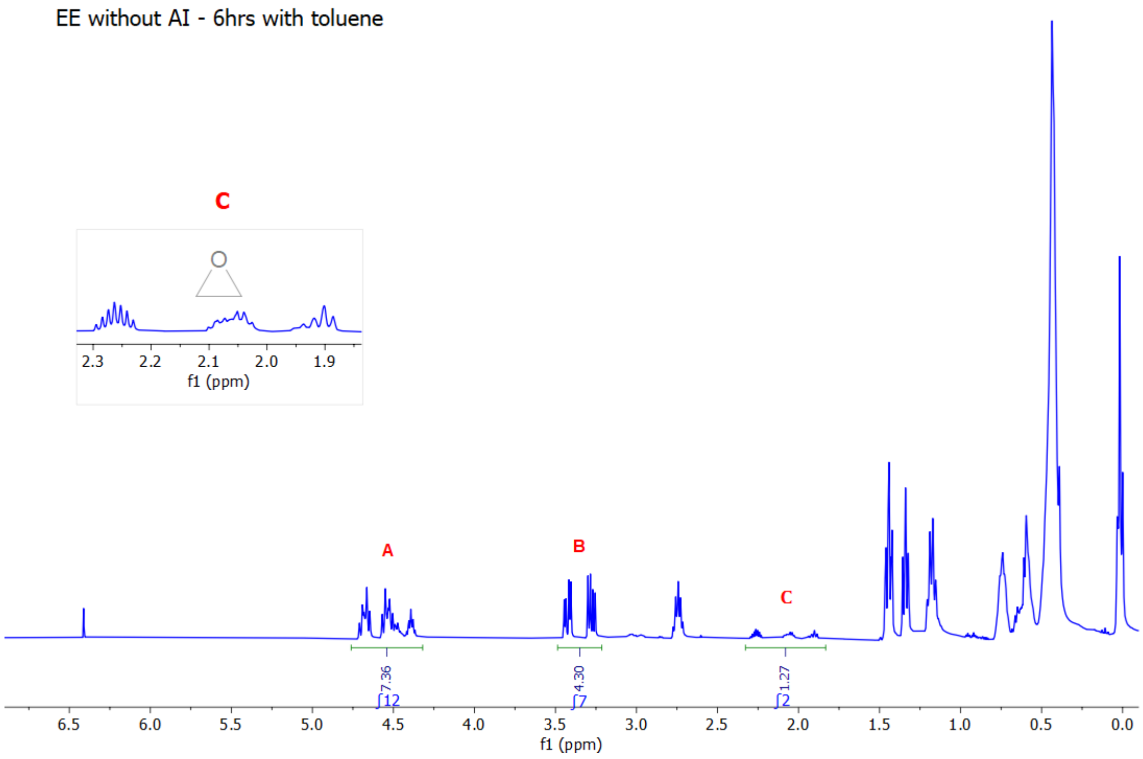

Figure S5: ^{1}H-NMR from the 6 hr enzymatic epoxidation reaction without AI with toluene.

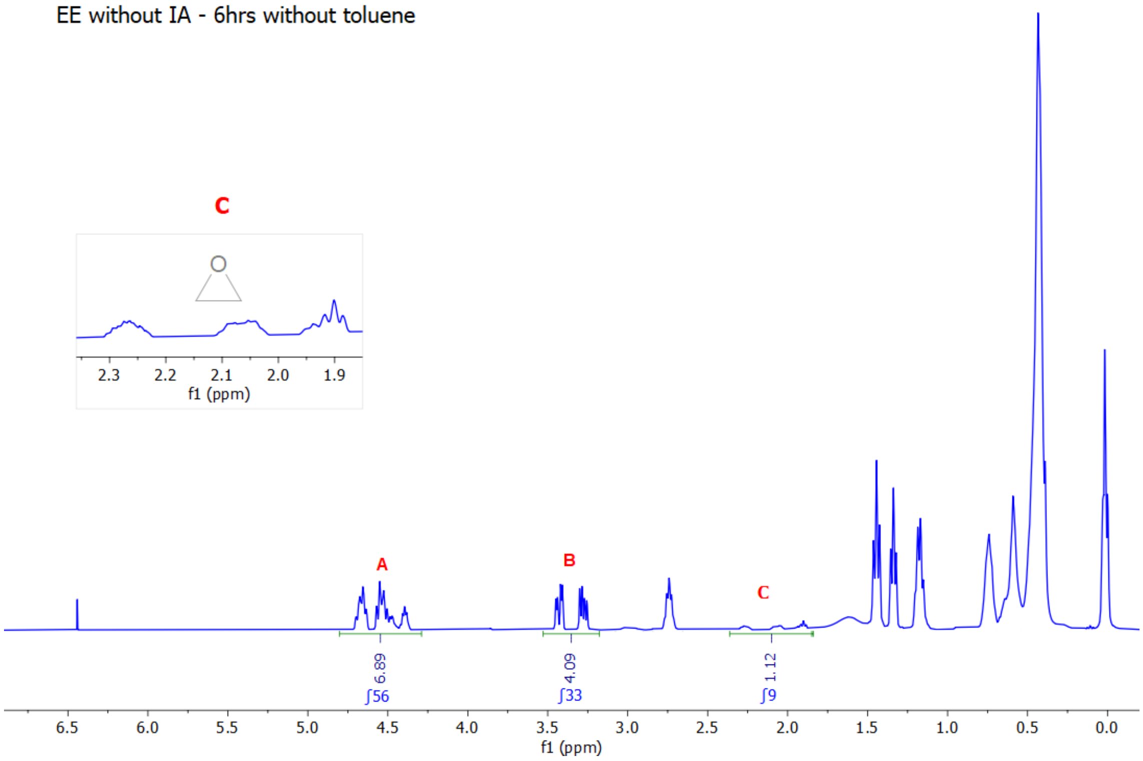

Figure S6: ^{1}H-NMR from the 6 hr enzymatic epoxidation reaction without AI without toluene.
